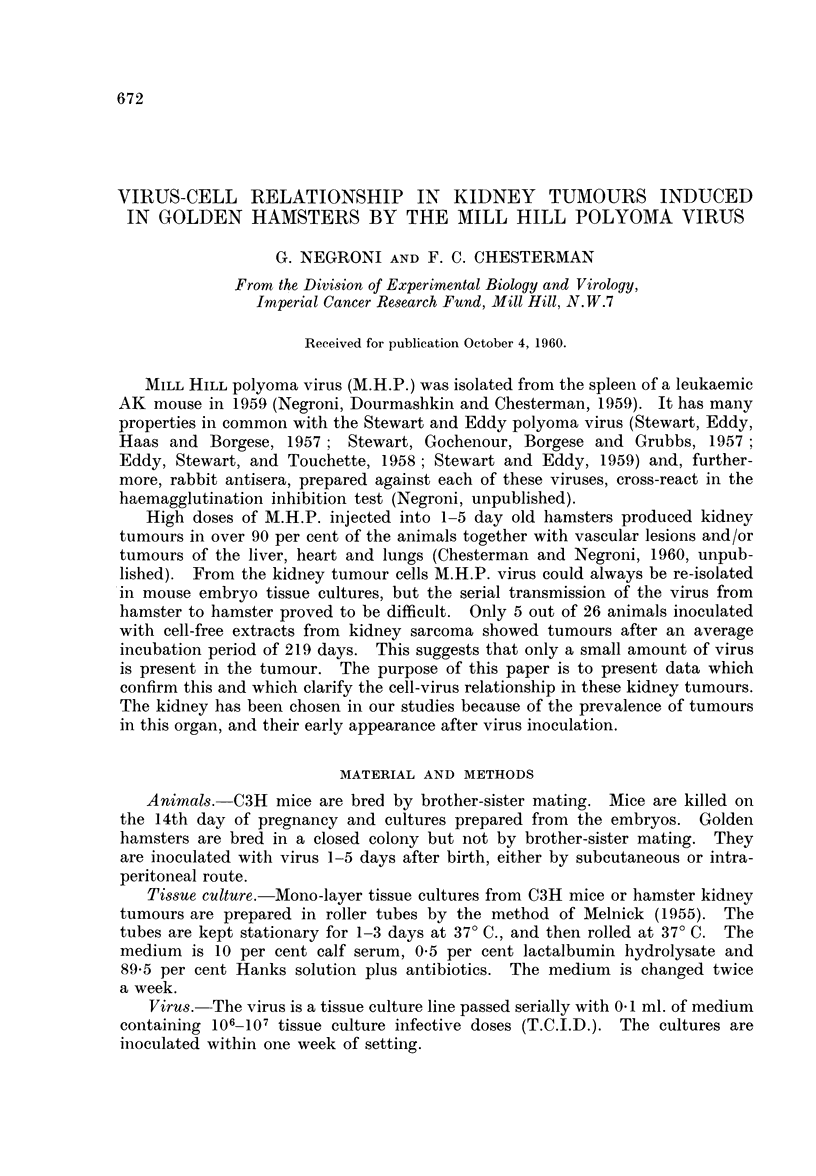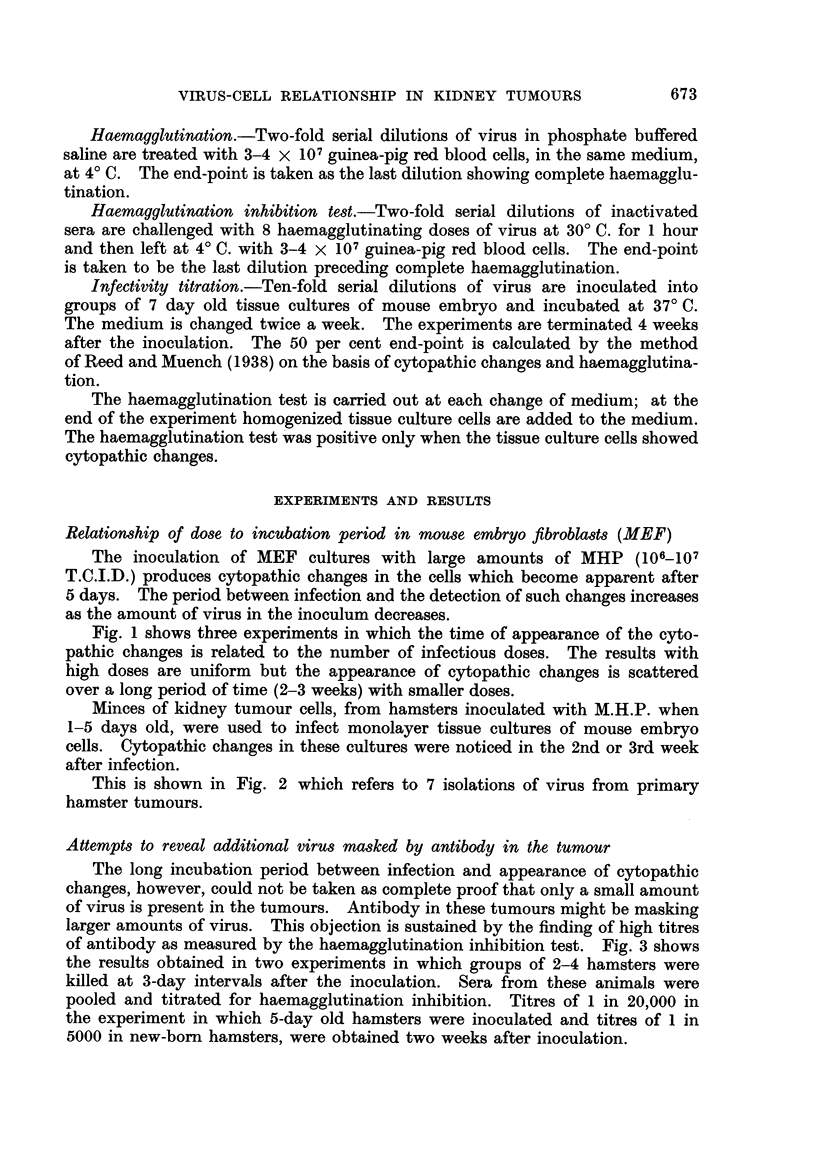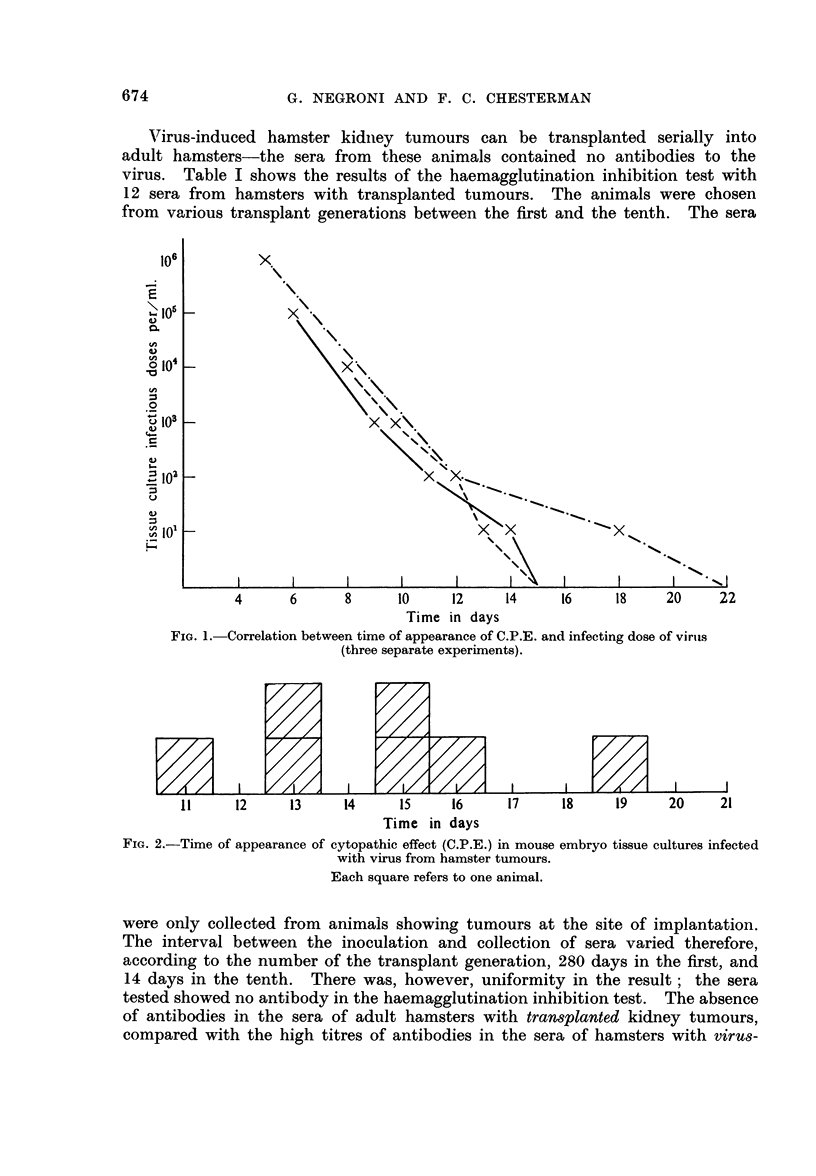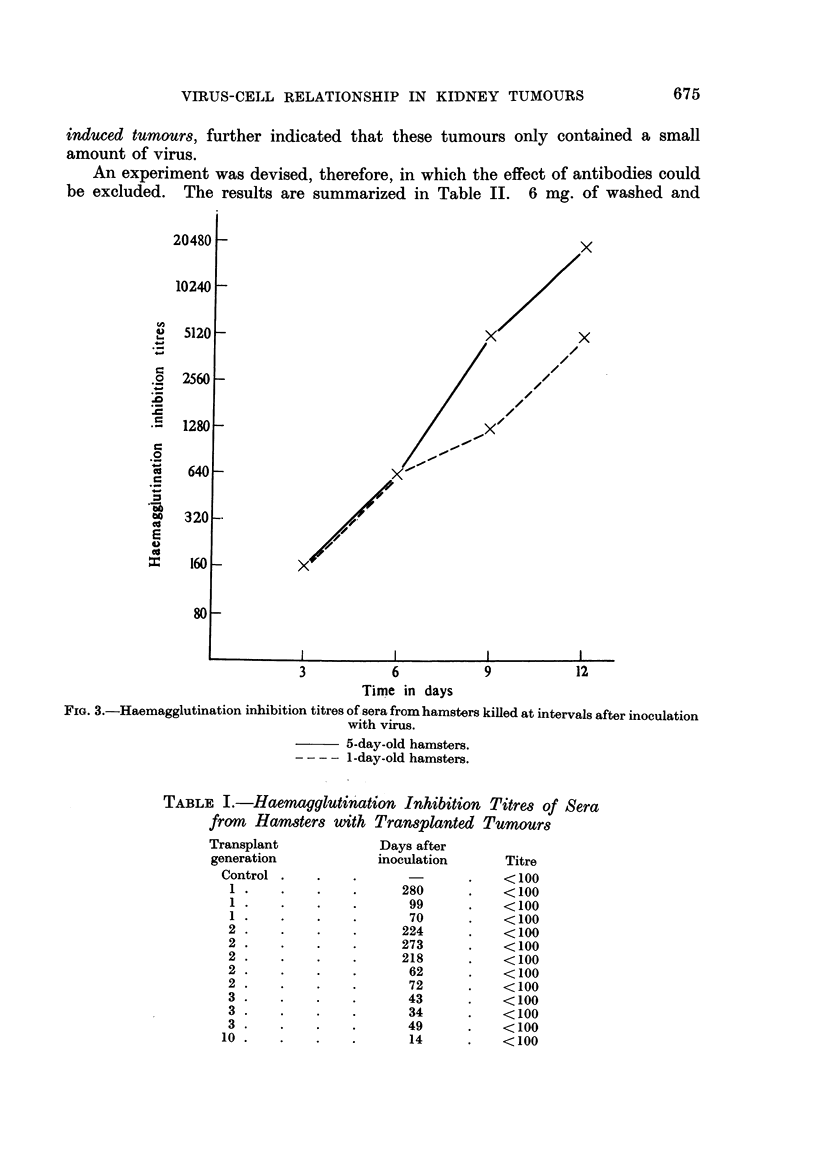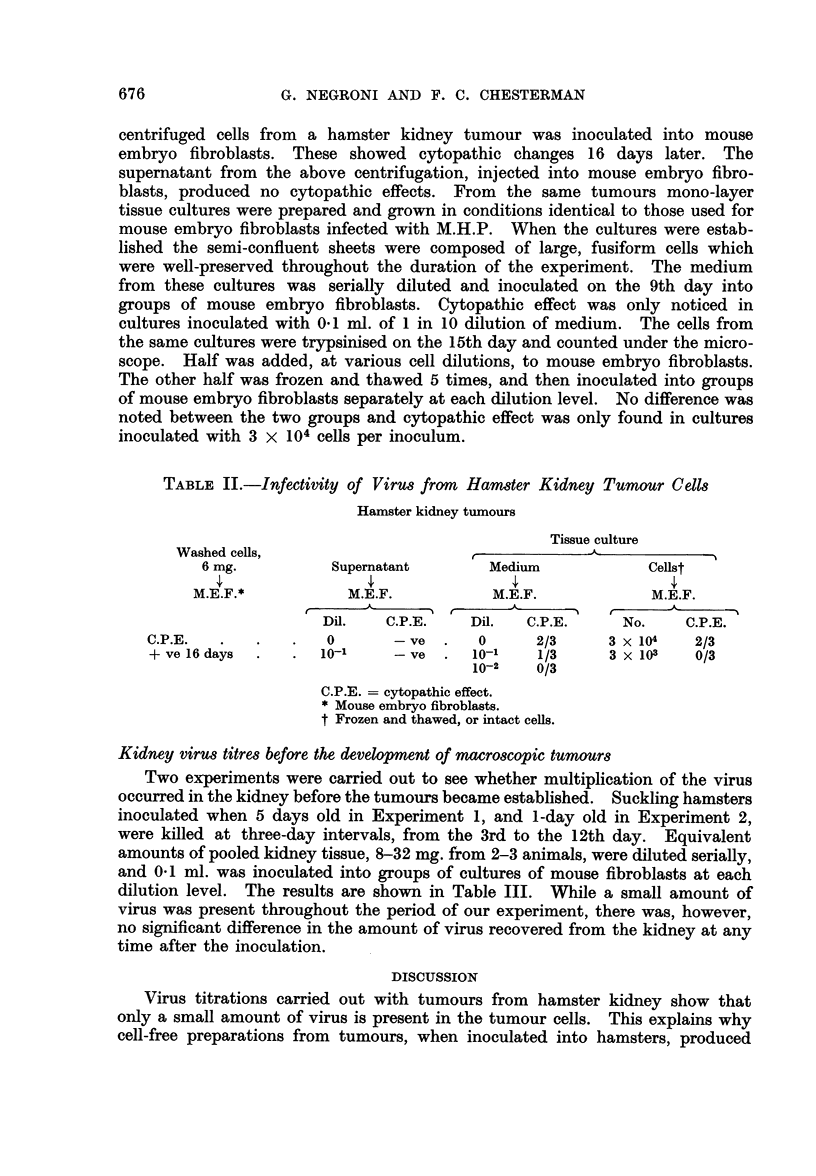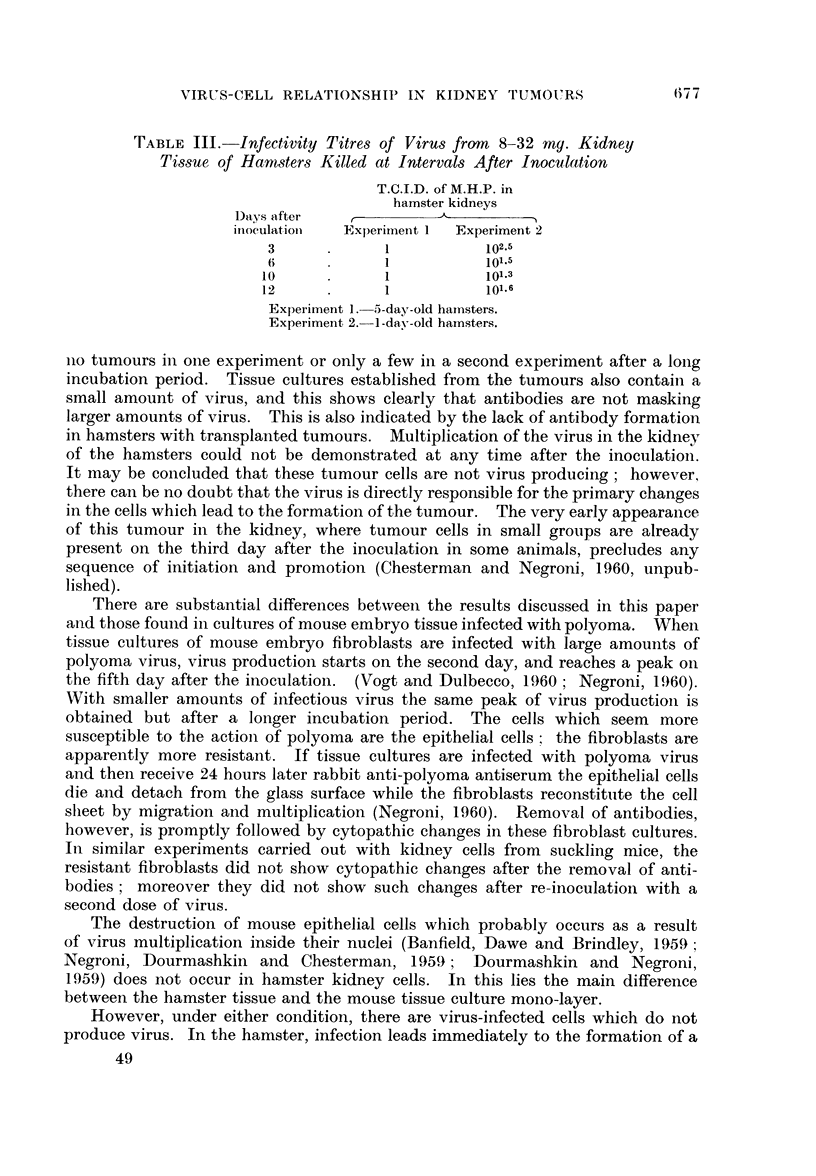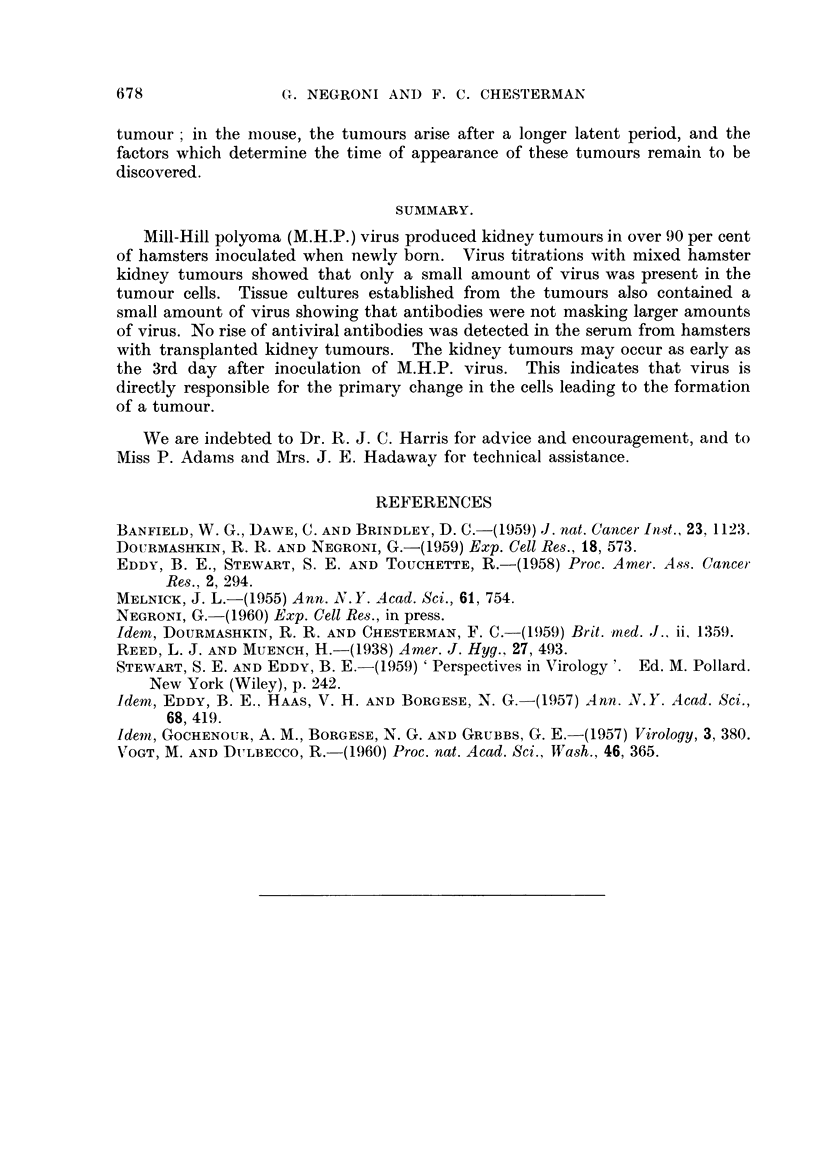# Virus-cell Relationship in Kidney Tumours Induced in Golden Hamsters by the Mill Hill Polyoma Virus

**DOI:** 10.1038/bjc.1960.75

**Published:** 1960-12

**Authors:** G. Negroni, F. C. Chesterman


### VIRUS-CELL RELATIONSHIP IN KIDNEY TUMOURS INDUCED IN GOLDEN HAMSTERS BY THE MILL HILL POLYOMA VIRUS

G. NEGRONI AND F. C. CHESTERMAN

*From the Division of Experimental Biology and Virology, Imperial Cancer Research Fund, Mill Hill, N.W.7*

Received for publication October 4, 1960.

MILL HILL polyoma virus (M.H.P.) was isolated from the spleen of a leukaemic AK mouse in 1959 (Negroni, Dourmashkin and Chesterman, 1959). It has many properties in common with the Stewart and Eddy polyoma virus (Stewart, Eddy, Haas and Borgese, 1957; Stewart, Gochenour, Borgese and Grubbs, 1957; Eddy, Stewart, and Touchette, 1958; Stewart and Eddy, 1959) and, furthermore, rabbit antisera, prepared against each of these viruses, cross-react in the haemagglutination inhibition test (Negroni, unpublished).

High doses of M.H.P. injected into 1–5 day old hamsters produced kidney tumours in over 90 per cent of the animals together with vascular lesions and/or tumours of the liver, heart and lungs (Chesterman and Negroni, 1960, unpublished). From the kidney tumour cells M.H.P. virus could always be re-isolated in mouse embryo tissue cultures, but the serial transmission of the virus from hamster to hamster proved to be difficult. Only 5 out of 26 animals inoculated with cell-free extracts from kidney sarcoma showed tumours after an average incubation period of 219 days. This suggests that only a small amount of virus is present in the tumour. The purpose of this paper is to present data which confirm this and which clarify the cell-virus relationship in these kidney tumours. The kidney has been chosen in our studies because of the prevalence of tumours in this organ, and their early appearance after virus inoculation.

### MATERIAL AND METHODS

*Animals.*—C3H mice are bred by brother-sister mating. Mice are killed on the 14th day of pregnancy and cultures prepared from the embryos. Golden hamsters are bred in a closed colony but not by brother-sister mating. They are inoculated with virus 1–5 days after birth, either by subcutaneous or intraperitoneal route.

*Tissue culture.*—Mono-layer tissue cultures from C3H mice or hamster kidney tumours are prepared in roller tubes by the method of Melnick (1955). The tubes are kept stationary for 1–3 days at 37° C., and then rolled at 37° C. The medium is 10 per cent calf serum, 0·5 per cent lactalbumin hydrolysate and 89·5 per cent Hanks solution plus antibiotics. The medium is changed twice a week.

*Virus.*—The virus is a tissue culture line passed serially with 0·1 ml. of medium containing $10^6$–$10^7$ tissue culture infective doses (T.C.I.D.). The cultures are inoculated within one week of setting.

*Haemagglutination.*—Two-fold serial dilutions of virus in phosphate buffered saline are treated with 3–4 $\times$ $10^7$ guinea-pig red blood cells, in the same medium, at 4° C. The end-point is taken as the last dilution showing complete haemagglutination.

*Haemagglutination inhibition test.*—Two-fold serial dilutions of inactivated sera are challenged with 8 haemagglutinating doses of virus at 30° C. for 1 hour and then left at 4° C. with 3–4 $\times$ $10^7$ guinea-pig red blood cells. The end-point is taken to be the last dilution preceding complete haemagglutination.

*Infectivity titration.*—Ten-fold serial dilutions of virus are inoculated into groups of 7 day old tissue cultures of mouse embryo and incubated at 37° C. The medium is changed twice a week. The experiments are terminated 4 weeks after the inoculation. The 50 per cent end-point is calculated by the method of Reed and Muench (1938) on the basis of cytopathic changes and haemagglutination.

The haemagglutination test is carried out at each change of medium; at the end of the experiment homogenized tissue culture cells are added to the medium. The haemagglutination test was positive only when the tissue culture cells showed cytopathic changes.

### EXPERIMENTS AND RESULTS

### *Relationship of dose to incubation period in mouse embryo fibroblasts (MEF)*

The inoculation of MEF cultures with large amounts of MHP ($10^6$–$10^7$ T.C.I.D.) produces cytopathic changes in the cells which become apparent after 5 days. The period between infection and the detection of such changes increases as the amount of virus in the inoculum decreases.

Fig. 1 shows three experiments in which the time of appearance of the cytopathic changes is related to the number of infectious doses. The results with high doses are uniform but the appearance of cytopathic changes is scattered over a long period of time (2–3 weeks) with smaller doses.

Minces of kidney tumour cells, from hamsters inoculated with M.H.P. when 1–5 days old, were used to infect monolayer tissue cultures of mouse embryo cells. Cytopathic changes in these cultures were noticed in the 2nd or 3rd week after infection.

This is shown in Fig. 2 which refers to 7 isolations of virus from primary hamster tumours.

### *Attempts to reveal additional virus masked by antibody in the tumour*

The long incubation period between infection and appearance of cytopathic changes, however, could not be taken as complete proof that only a small amount of virus is present in the tumours. Antibody in these tumours might be masking larger amounts of virus. This objection is sustained by the finding of high titres of antibody as measured by the haemagglutination inhibition test. Fig. 3 shows the results obtained in two experiments in which groups of 2–4 hamsters were killed at 3-day intervals after the inoculation. Sera from these animals were pooled and titrated for haemagglutination inhibition. Titres of 1 in 20,000 in the experiment in which 5-day old hamsters were inoculated and titres of 1 in 5000 in new-born hamsters, were obtained two weeks after inoculation.

Virus-induced hamster kidney tumours can be transplanted serially into adult hamsters—the sera from these animals contained no antibodies to the virus. Table I shows the results of the haemagglutination inhibition test with 12 sera from hamsters with transplanted tumours. The animals were chosen from various transplant generations between the first and the tenth. The sera

Fig. 1.—Correlation between time of appearance of C.P.E. and infecting dose of virus (three separate experiments).

Fig. 2.—Time of appearance of cytopathic effect (C.P.E.) in mouse embryo tissue cultures infected with virus from hamster tumours.

Each square refers to one animal.

were only collected from animals showing tumours at the site of implantation. The interval between the inoculation and collection of sera varied therefore, according to the number of the transplant generation, 280 days in the first, and 14 days in the tenth. There was, however, uniformity in the result ; the sera tested showed no antibody in the haemagglutination inhibition test. The absence of antibodies in the sera of adult hamsters with *transplanted* kidney tumours, compared with the high titres of antibodies in the sera of hamsters with *virus-*

*induced tumours*, further indicated that these tumours only contained a small amount of virus.

An experiment was devised, therefore, in which the effect of antibodies could be excluded. The results are summarized in Table II. 6 mg. of washed and

FIG. 3.—Haemagglutination inhibition titres of sera from hamsters killed at intervals after inoculation with virus.

——— 5-day-old hamsters.
– – – – 1-day-old hamsters.

TABLE I.—*Haemagglutination Inhibition Titres of Sera from Hamsters with Transplanted Tumours*

| Transplant generation | Days after inoculation | Titre |
|---|---|---|
| Control | — | <100 |
| 1 | 280 | <100 |
| 1 | 99 | <100 |
| 1 | 70 | <100 |
| 2 | 224 | <100 |
| 2 | 273 | <100 |
| 2 | 218 | <100 |
| 2 | 62 | <100 |
| 2 | 72 | <100 |
| 3 | 43 | <100 |
| 3 | 34 | <100 |
| 3 | 49 | <100 |
| 10 | 14 | <100 |

centrifuged cells from a hamster kidney tumour was inoculated into mouse embryo fibroblasts. These showed cytopathic changes 16 days later. The supernatant from the above centrifugation, injected into mouse embryo fibroblasts, produced no cytopathic effects. From the same tumours mono-layer tissue cultures were prepared and grown in conditions identical to those used for mouse embryo fibroblasts infected with M.H.P. When the cultures were established the semi-confluent sheets were composed of large, fusiform cells which were well-preserved throughout the duration of the experiment. The medium from these cultures was serially diluted and inoculated on the 9th day into groups of mouse embryo fibroblasts. Cytopathic effect was only noticed in cultures inoculated with 0·1 ml. of 1 in 10 dilution of medium. The cells from the same cultures were trypsinised on the 15th day and counted under the microscope. Half was added, at various cell dilutions, to mouse embryo fibroblasts. The other half was frozen and thawed 5 times, and then inoculated into groups of mouse embryo fibroblasts separately at each dilution level. No difference was noted between the two groups and cytopathic effect was only found in cultures inoculated with $3 \times 10^4$ cells per inoculum.

TABLE II.—*Infectivity of Virus from Hamster Kidney Tumour Cells*

Hamster kidney tumours

| Washed cells, 6 mg. ↓ M.E.F.* | Supernatant ↓ M.E.F. | | Tissue culture: Medium ↓ M.E.F. | | Tissue culture: Cells† ↓ M.E.F. | |
|---|---|---|---|---|---|---|
| | Dil. | C.P.E. | Dil. | C.P.E. | No. | C.P.E. |
| C.P.E. | 0 | − ve | 0 | 2/3 | $3 \times 10^4$ | 2/3 |
| + ve 16 days | $10^{-1}$ | − ve | $10^{-1}$ | 1/3 | $3 \times 10^3$ | 0/3 |
| | | | $10^{-2}$ | 0/3 | | |

C.P.E. = cytopathic effect.
* Mouse embryo fibroblasts.
† Frozen and thawed, or intact cells.

*Kidney virus titres before the development of macroscopic tumours*

Two experiments were carried out to see whether multiplication of the virus occurred in the kidney before the tumours became established. Suckling hamsters inoculated when 5 days old in Experiment 1, and 1-day old in Experiment 2, were killed at three-day intervals, from the 3rd to the 12th day. Equivalent amounts of pooled kidney tissue, 8–32 mg. from 2–3 animals, were diluted serially, and 0·1 ml. was inoculated into groups of cultures of mouse fibroblasts at each dilution level. The results are shown in Table III. While a small amount of virus was present throughout the period of our experiment, there was, however, no significant difference in the amount of virus recovered from the kidney at any time after the inoculation.

### DISCUSSION

Virus titrations carried out with tumours from hamster kidney show that only a small amount of virus is present in the tumour cells. This explains why cell-free preparations from tumours, when inoculated into hamsters, produced

TABLE III.—*Infectivity Titres of Virus from 8–32 mg. Kidney Tissue of Hamsters Killed at Intervals After Inoculation*

| Days after inoculation | T.C.I.D. of M.H.P. in hamster kidneys: Experiment 1 | Experiment 2 |
|---|---|---|
| 3 | 1 | $10^{2.5}$ |
| 6 | 1 | $10^{1.5}$ |
| 10 | 1 | $10^{1.3}$ |
| 12 | 1 | $10^{1.6}$ |

Experiment 1.—5-day-old hamsters.
Experiment 2.—1-day-old hamsters.

no tumours in one experiment or only a few in a second experiment after a long incubation period. Tissue cultures established from the tumours also contain a small amount of virus, and this shows clearly that antibodies are not masking larger amounts of virus. This is also indicated by the lack of antibody formation in hamsters with transplanted tumours. Multiplication of the virus in the kidney of the hamsters could not be demonstrated at any time after the inoculation. It may be concluded that these tumour cells are not virus producing ; however, there can be no doubt that the virus is directly responsible for the primary changes in the cells which lead to the formation of the tumour. The very early appearance of this tumour in the kidney, where tumour cells in small groups are already present on the third day after the inoculation in some animals, precludes any sequence of initiation and promotion (Chesterman and Negroni, 1960, unpublished).

There are substantial differences between the results discussed in this paper and those found in cultures of mouse embryo tissue infected with polyoma. When tissue cultures of mouse embryo fibroblasts are infected with large amounts of polyoma virus, virus production starts on the second day, and reaches a peak on the fifth day after the inoculation. (Vogt and Dulbecco, 1960 ; Negroni, 1960). With smaller amounts of infectious virus the same peak of virus production is obtained but after a longer incubation period. The cells which seem more susceptible to the action of polyoma are the epithelial cells ; the fibroblasts are apparently more resistant. If tissue cultures are infected with polyoma virus and then receive 24 hours later rabbit anti-polyoma antiserum the epithelial cells die and detach from the glass surface while the fibroblasts reconstitute the cell sheet by migration and multiplication (Negroni, 1960). Removal of antibodies, however, is promptly followed by cytopathic changes in these fibroblast cultures. In similar experiments carried out with kidney cells from suckling mice, the resistant fibroblasts did not show cytopathic changes after the removal of antibodies ; moreover they did not show such changes after re-inoculation with a second dose of virus.

The destruction of mouse epithelial cells which probably occurs as a result of virus multiplication inside their nuclei (Banfield, Dawe and Brindley, 1959 ; Negroni, Dourmashkin and Chesterman, 1959 ; Dourmashkin and Negroni, 1959) does not occur in hamster kidney cells. In this lies the main difference between the hamster tissue and the mouse tissue culture mono-layer.

However, under either condition, there are virus-infected cells which do not produce virus. In the hamster, infection leads immediately to the formation of a tumour ; in the mouse, the tumours arise after a longer latent period, and the factors which determine the time of appearance of these tumours remain to be discovered.

SUMMARY.

Mill-Hill polyoma (M.H.P.) virus produced kidney tumours in over 90 per cent of hamsters inoculated when newly born. Virus titrations with mixed hamster kidney tumours showed that only a small amount of virus was present in the tumour cells. Tissue cultures established from the tumours also contained a small amount of virus showing that antibodies were not masking larger amounts of virus. No rise of antiviral antibodies was detected in the serum from hamsters with transplanted kidney tumours. The kidney tumours may occur as early as the 3rd day after inoculation of M.H.P. virus. This indicates that virus is directly responsible for the primary change in the cells leading to the formation of a tumour.

We are indebted to Dr. R. J. C. Harris for advice and encouragement, and to Miss P. Adams and Mrs. J. E. Hadaway for technical assistance.

REFERENCES

BANFIELD, W. G., DAWE, C. AND BRINDLEY, D. C.—(1959) *J. nat. Cancer Inst.*, **23**, 1123.

DOURMASHKIN, R. R. AND NEGRONI, G.—(1959) *Exp. Cell Res.*, **18**, 573.

EDDY, B. E., STEWART, S. E. AND TOUCHETTE, R.—(1958) *Proc. Amer. Ass. Cancer Res.*, **2**, 294.

MELNICK, J. L.—(1955) *Ann. N.Y. Acad. Sci.*, **61**, 754.

NEGRONI, G.—(1960) *Exp. Cell Res.*, in press.

*Idem*, DOURMASHKIN, R. R. AND CHESTERMAN, F. C.—(1959) *Brit. med. J.*, ii, 1359.

REED, L. J. AND MUENCH, H.—(1938) *Amer. J. Hyg.*, **27**, 493.

STEWART, S. E. AND EDDY, B. E.—(1959) ' Perspectives in Virology '. Ed. M. Pollard. New York (Wiley), p. 242.

*Idem*, EDDY, B. E., HAAS, V. H. AND BORGESE, N. G.—(1957) *Ann. N.Y. Acad. Sci.*, **68**, 419.

*Idem*, GOCHENOUR, A. M., BORGESE, N. G. AND GRUBBS, G. E.—(1957) *Virology*, **3**, 380.

VOGT, M. AND DULBECCO, R.—(1960) *Proc. nat. Acad. Sci., Wash.*, **46**, 365.